# Context-dependent functions of insulin, IGF, and GLP signaling in inflammatory bowel disease: linking metabolism, immunity, and intestinal homeostasis

**DOI:** 10.3389/fimmu.2026.1970634

**Published:** 2026-10-21

**Authors:** Ezzuddin Abuhussein, Yukihiro Yamaguchi, Dhirendra K. Singh, Chiara Elena Orahovats, Keita Nishiyama, Koichi Tsuboi, Lei Huang

**Affiliations:** 1 Children’s Research Institute, University of North Carolina at Chapel Hill, Chapel Hill, NC, United States; 2 Department of Pediatrics, University of North Carolina at Chapel Hill, Chapel Hill, NC, United States; 3 Department of Rheumatology and Inflammation Research, University of Gothenburg, Gothenburg, Sweden; 4 Dietrich School of Arts & Sciences, University of Pittsburgh, Pittsburgh, PA, United States; 5 Laboratory of Animal Food Function, School of Agricultural Science, Tohoku University, Sendai, Japan; 6 Department of Pediatric Surgery, School of Medicine Juntendo University, Tokyo, Japan; 7 Faculty of Medical Sciences, Newcastle University, Newcastle upon Tyne, United Kingdom

**Keywords:** inflammatory bowel disease (IBD), insulin, insulin-like growth factor (IGF), IGF-binding protein (IGFBP), glucagon-like peptide (GLP)

## Abstract

Inflammatory bowel disease (IBD), encompassing Crohn’s disease and ulcerative colitis, is a chronic relapsing inflammatory disorder of the gastrointestinal tract characterized by dysregulated immune responses, impaired epithelial barrier function, and alterations in the gut microbiota. Although considerable progress has been made in understanding the immunopathogenesis of IBD, increasing evidence indicates that metabolic signaling pathways are integral regulators of intestinal inflammation, tissue repair, and disease progression. Among these, insulin, insulin-like growth factors (IGFs), and glucagon-like peptides (GLPs) constitute an interconnected immunometabolic network that links nutrient sensing, energy metabolism, epithelial regeneration, and immune homeostasis. Insulin signaling plays context-dependent roles in IBD by regulating glucose metabolism, epithelial repair, inflammatory responses, and interactions with the gut microbiota and bile acid metabolism. While insulin promotes mucosal healing and barrier restoration in intestinal epithelial cells, excessive activation of insulin signaling in immune cells may exacerbate chronic inflammation through epigenetic and cytokine-mediated mechanisms. The IGF axis is also profoundly altered during active IBD, with systemic suppression of IGF-1 and IGFBP-3 accompanying disease activity, whereas local IGF signaling promotes epithelial regeneration, wound healing, and immune regulation. In addition, IGF-binding proteins (IGFBPs) function as active modulators of inflammation, fibrosis, and colitis-associated colorectal cancer in an isoform-specific manner. GLP signaling further integrates metabolism with intestinal immunity; GLP-1 receptor agonists exhibit anti-inflammatory, metabolic, and microbiota-modulating effects, whereas GLP-2 primarily enhances epithelial proliferation, barrier integrity, and mucosal repair. Emerging preclinical and observational clinical studies suggest that GLP-based therapies may improve disease outcomes, although prospective randomized trials are still required. This review summarizes current knowledge regarding the biological functions and molecular mechanisms of insulin, IGFs, IGFBPs, and GLPs in IBD, highlighting their coordinated roles in regulating metabolism, immunity, epithelial homeostasis, and tissue remodeling. A better understanding of these interconnected signaling pathways may facilitate the development of novel immunometabolic therapeutic strategies aimed at promoting mucosal healing, limiting chronic inflammation, and improving long-term outcomes in patients with IBD.

## 1 Introduction

Inflammatory bowel disease (IBD), comprising Crohn’s disease (CD) and ulcerative colitis (UC), is characterized by chronic and relapsing intestinal inflammation resulting from complex interactions among immune dysregulation, epithelial barrier dysfunction, the gut microbiota, genetic susceptibility, and environmental factors (1). Although IBD is traditionally viewed primarily as an immune-mediated disorder, increasing evidence indicates that intestinal inflammation is closely connected to systemic metabolic regulation (2). Alterations in glucose metabolism, insulin sensitivity, adipose tissue function, and nutrient-sensing pathways occur in subsets of patients with IBD and may influence disease activity, tissue repair, and long-term complications (3, 4). Conversely, chronic intestinal inflammation can affect systemic metabolic homeostasis, suggesting that the relationship between inflammation and metabolism is bidirectional (5).

Among the signaling pathways that integrate metabolic and inflammatory processes, insulin, insulin-like growth factors (IGF-1 and IGF-2), and glucagon-like peptides (GLP-1 and GLP-2) are of particular interest (**Figure 1**). The ingestion of glucose and nutrients induces the secretion of insulin from the pancreas as well as GLP-1 and GLP-2 from the intestine (6). While GLP-1 mainly potentiates glucose-dependent insulin secretion and reduces glucagon secretion, GLP-2 is involved in intestinal adaptation, modulation of nutrient absorption, and regulation of hepatic glucose metabolism (7–9). Insulin facilitates glucose uptake in peripheral tissues and modulates the IGF system, for example, by regulating hepatic production of IGF-1 and IGF-1 bioavailability (10–12). In turn, IGF-1 and IGF-2 exhibit both metabolic and growth factor activities similar to insulin but act through different sets of insulin and IGF receptors (13–15). GLP signaling might also exert effects on the IGF system, such as the regulation of IGF-binding protein (IGFBP)-1 expression by GLP-1 (16) or the intestinal IGF-1-trophic effects mediated by GLP-2 (17).

**Figure 1:**
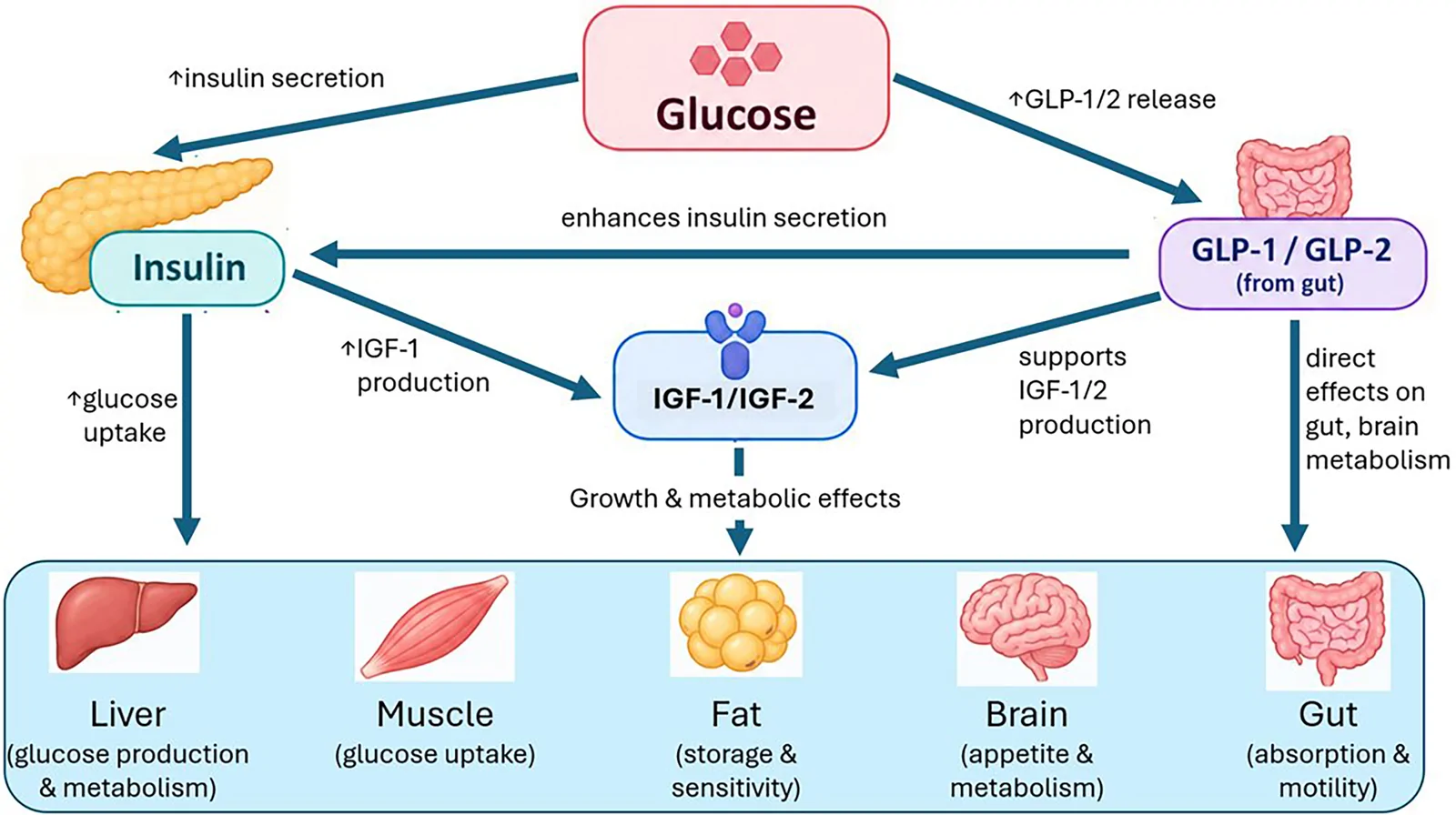
Interconnected glucose–insulin–IGF-GLP signaling network. Glucose availability promotes both pancreatic insulin secretion and intestinal release of GLP-1 and GLP-2. GLP-1 is involved in promoting glucose-dependent insulin secretion and in suppressing glucagon release, while GLP-2 is responsible for intestinal adaptation, nutrient absorption, and the control of hepatic glucose metabolism. Insulin is involved in increasing peripheral glucose uptake and in interacting with the IGF system, which includes controlling the production of hepatic IGF-1 and its bioavailability. IGF-1 and IGF-2 have similar roles to those of insulin in terms of metabolic and growth promoting effects; however, they use different combinations of insulin and IGF receptors to transmit signals. GLP signaling can also influence the IGF axis, including GLP-1-mediated effects on IGFBP-1 and GLP-2-dependent intestinal trophic effects involving IGF-1.

Insulin signaling regulates glucose utilization and cellular metabolism while also influencing epithelial survival, immune-cell function, and inflammatory responses (18, 19).

Insulin and IGF-1 also directly influence immune-cell metabolism, an interaction that is particularly relevant to IBD. In activated CD4^+^ T cells, IGF-1R expression increases, and IGF-1 has a particularly strong effect on Th17 cells, increasing their metabolism and IL-17 production while reducing mitochondrial membrane potential and mitochondrial ROS. Interestingly, both IR and IGF-1R were required for some of these mitochondrial effects, suggesting participation of hybrid receptors (20). These findings indicate that the insulin/IGF-1 metabolic network extends into immune regulation and intestinal inflammation.

The clinical relevance of these pathways is further highlighted by the frequent coexistence of IBD with metabolic disorders, including obesity, insulin resistance, and diabetes mellitus (DM). The association between IBD and DM appears to be complex and may involve chronic inflammation, altered insulin signaling, intestinal barrier dysfunction, gut microbiota, and shared environmental or genetic factors (21, 22). At the same time, metabolic therapies that modify insulin- or GLP-related signaling are increasingly being investigated for their potential effects on intestinal inflammation and tissue repair (19, 23, 24). Despite growing evidence, the effects of IBD on systemic insulin sensitivity and related metabolic pathways have received less integrated consideration. A framework that considers both directions of this relationship may therefore provide a more comprehensive understanding of how metabolic and inflammatory signals interact during intestinal disease.

In this review, we first synthesize current evidence on the context-dependent functions of insulin, IGF, IGFBP, and GLP signaling in IBD. We then consider how IBD and its associated inflammatory and metabolic disturbances alter systemic insulin signaling. Finally, we integrate these findings into a bidirectional immunometabolic framework and discuss the therapeutic implications and remaining challenges for targeting these pathways in IBD.

## 2 Effects of insulin on IBD

In IBD and associated metabolic disorders, glucose metabolism and insulin signaling are increasingly recognized as critical factors in the regulation of intestinal inflammation, epithelial homeostasis, and tumorigenesis, although their roles vary depending on the biological context. Early clinical metabolic studies demonstrated that systemic insulin sensitivity and glucose metabolism, assessed via the hyperinsulinemic-euglycemic clamp technique, remained largely preserved in patients with CD and UC who were in remission; specifically, levels of systemic glucose uptake and oxidation were comparable to those of healthy controls (25). These findings suggest that insulin resistance is not an inherent feature of IBD in remission but is instead closely linked to inflammatory activity. However, subsequent larger-scale clinical analyses revealed disease heterogeneity. Notably, increased insulin resistance (HOMA-IR) was reported in UC but not in CD, suggesting the existence of subtype-specific metabolic alterations potentially associated with persistent low-grade inflammation (26).

At a mechanistic level, insulin signaling interacts bidirectionally with inflammatory pathways. Inflammatory cytokines, such as TNFα, can modulate the activity of metabolic enzymes in the intestine. For instance, insulin and inflammatory mediators increase the activity of sulfotransferases (SULT1A1 and SULT1A3) in colonic epithelial cells, thereby promoting the bioactivation of dietary carcinogens and potentially linking metabolic and inflammatory states to colorectal cancer risk (27). Conversely, insulin has been proposed to exert anti-inflammatory effects, such as the inhibition of TNFα production, oxidative stress, and leukocyte adhesion, leading to early hypotheses that glucose-insulin-potassium therapy might be useful for treating IBD (28). More recent experimental evidence supports these anti-inflammatory effects, demonstrating that low-dose insulin can alleviate colitis in mice. The underlying mechanisms involve remodeling of the gut microbiota and increased production of lithocholic acid, which activates the bile acid receptor TGR5 and suppresses polarization toward pro-inflammatory M1 macrophages (29).

Importantly, insulin signaling in the intestinal mucosa exhibits strong tissue and cell-type specificity. In the epithelial compartment, insulin receptor signaling appears to play a protective role. Indeed, in models of chemically induced colitis, rectal administration of insulin reduces inflammation severity, promotes mucosal healing, decreases COX-2 expression, and suppresses colitis-associated tumorigenesis. These findings suggest that insulin signaling plays a beneficial role in epithelial repair and the restoration of barrier function (30). In contrast, activation of mucosal insulin receptor signaling in immune cells can exacerbate inflammation. Intrarectal insulin administration has been shown to worsen colitis by promoting the EZH2-dependent differentiation of tissue-resident memory T cells (TRM), increasing the production of TNFα and IL-17, and amplifying chronic intestinal inflammation in mice (31). These findings indicate that insulin signaling in epithelial cells and immune cells can lead to opposing immunological outcomes, depending on the cellular context and downstream epigenetic regulation.

Notably, both of these effects were produced using the same local route of insulin delivery, rectal or intrarectal instillation, indicating that the divergence reflects the cell population reached by insulin rather than a difference in administration route (30, 31). The two studies also differed in disease model and phase: the protective epithelial effect was observed in a chemically induced model of colitis-associated tumorigenesis, whereas the pathogenic, EZH2/TRM-mediated effect was observed specifically in chronic dextran sodium sulfate (DSS)-induced colitis. Although this raises the possibility that disease chronicity shifts the balance toward the T-cell-mediated pathway, the two models have not been directly compared, and this interpretation remains hypothetical.

Furthermore, epigenetic regulation integrates insulin-related pathways into the control mechanisms of intestinal inflammation. In UC mice, the CCCTC-binding factor (CTCF) recruits EZH2 to the promoter region of IGFBP-5. This induces H3K27me3-mediated transcriptional repression, thereby exacerbating epithelial injury through activation of NF-κB-driven inflammatory responses (32). These findings highlight a broader link between chromatin remodeling mechanisms, which govern epithelial resilience and inflammatory status, and insulin/IGF-related signaling.

Beyond inflammation, evidence from both clinical and experimental studies suggests that insulin signaling plays a role in the development of colorectal cancer. In patients with type 2 diabetes, insulin therapy was associated with a higher colorectal adenoma detection rate compared with metformin monotherapy or combination therapy, suggesting that exposure to exogenous insulin may contribute to cell proliferation and tumor promotion (33). Furthermore, in mouse experimental models of colitis-associated cancer, enhanced insulin and IGF-1 signaling have been shown to correlate with visceral fat accumulation, increased leptin production, and the activation of downstream metabolic pathways in adipose tissue; collectively, these factors contribute to systemic metabolic inflammation that promotes tumorigenesis (34). Concurrently, as noted above, insulin and inflammatory cytokines may enhance the sulfotransferase-mediated activation of dietary carcinogen precursors in colonic epithelial cells, indicating an additional mechanistic link between metabolic signaling and colorectal cancer risk (27). Beyond these local, cell-type-specific effects, insulin can also act systemically to limit colitis through a distinct, microbiome-dependent pathway. Low-dose insulin given intraperitoneally alleviates both acute and chronic colitis in mice by promoting gut microbial production of the secondary bile acid lithocholic acid, which activates TGR5 and suppresses pro-inflammatory macrophage polarization; this protective effect is abolished by antibiotic-induced depletion of the microbiota and restored by fecal microbiota transplantation, confirming its dependence on the host microbiome (29). Taken together, the apparently conflicting effects of insulin on the intestine described above can be reconciled by considering, jointly, the cell population and anatomical compartment reached (epithelium versus mucosal T cells), the route and dose of delivery (local versus systemic, low- versus high-dose), the phase of disease, and the status of the gut microbiota, rather than any single factor in isolation. Whether host metabolic phenotype, such as obesity or pre-existing insulin resistance, further modifications of these context-dependent effects have not yet been systematically examined and represent an important gap for future studies. Based on experimental findings regarding microbiome-dependent alterations in bile acid metabolism and macrophage regulation, strategies involving colon-targeted or low-dose insulin administration are being investigated as a means to harness local anti-inflammatory effects while minimizing systemic metabolic impacts (29, 35).

## 3 IGF axis in IBD: from systemic suppression to local repair and pathological remodeling

The IGF system, comprising IGF-1, IGF-2, IGF receptors, and IGFBPs, is an important component of the metabolic and tissue-regulatory network that becomes profoundly altered during intestinal inflammation. Evidence from patients with IBD and experimental models indicates that inflammation affects the IGF axis at both systemic and local levels. A consistent clinical observation is that circulating IGF-1 and IGFBP-3 decrease during active disease, likely reflecting the interaction between inflammation, nutritional status, and altered anabolic signaling. In contrast, IGF signaling within the intestinal mucosa can remain active or become locally induced and may promote epithelial regeneration and immune resolution. However, persistent or excessive activation in particular cellular compartments may also contribute to fibrosis and carcinogenesis. Thus, the biological effects of the IGF axis in IBD depend strongly on the tissue compartment, cellular context, disease stage, and specific IGF or IGFBP involved.

### 3.1 Systemic IGF dysregulation during active IBD

In adult patients with active IBD, circulating IGF-1 and IGFBP-3 levels are significantly lower than those in healthy controls, whereas inflammatory cytokines such as IL-6 are increased (36). Similar changes have been reported in pediatric IBD, in which IGF-1 and IGFBP-3 increase during remission and show an inverse relationship with disease activity (37, 38). In both CD and UC, lower circulating IGF-1 concentrations were associated with higher CRP levels and greater disease activity, supporting an association between suppression of the IGF axis and systemic inflammatory burden (38, 39). Importantly, these abnormalities involve not only total circulating IGF concentrations but also the bioavailability of biologically active IGF. In patients with moderate-to-severe IBD, intact IGFBP-3 is reduced, potentially reflecting increased proteolytic cleavage and further limiting IGF activity (40).

The systemic suppression of the IGF axis is closely linked to the metabolic consequences of chronic intestinal inflammation. IGF-1, IGF-2, and IGFBP-3 correlate with albumin, CRP, IL-6, and other measures of disease activity, suggesting coordinated regulation of growth-factor signaling, nutritional status, and inflammation (39). IGF-2 is also associated with IL-6 during active disease, indicating that inflammatory regulation extends beyond IGF-1 alone (39). Changes in the IGFBP profile may further contribute to reduced IGF bioavailability, with active disease characterized by increased IGFBP-2 and decreased IGFBP-3 (41). These systemic alterations have clinically relevant consequences. In pediatric IBD, low IGF-1 levels are associated with elevated inflammatory cytokines, fatigue, impaired growth, and reduced quality of life (42), whereas in adults, abnormalities of the IGF axis are associated with catabolic complications including bone loss and muscle atrophy (43, 44). Collectively, these observations suggest that suppression of systemic IGF signaling is closely integrated with the inflammatory and catabolic state of active IBD rather than representing an isolated endocrine abnormality. However, these circulating measurements are observational associations that are influenced by nutritional status and IGFBP proteolysis. They do not establish a causal role for IGFBPs in intestinal inflammation or repair, nor do they indicate IGFBP activity within the inflamed mucosa, which is considered in Section 3.2.

### 3.2 Local IGF/IGFBP signaling in intestinal repair and immune regulation

Although systemic IGF activity is reduced during active IBD, experimental studies indicate that IGF signaling within the intestinal tissue can have distinct, and often protective, effects. In chemically induced colitis, IGF-1 administration promotes mucosal healing and structural recovery, including increased goblet-cell numbers, reduced submucosal thickening, restoration of epithelial integrity, and improvement of mucosal architecture (45). Mechanistic studies further demonstrate that IGF-1 promotes epithelial proliferation and regeneration through a β-arrestin-2-dependent ERK signaling pathway, indicating that downstream scaffold proteins are required for an effective regenerative response (46). In intestinal epithelial cells, IGF-1 can also activate IGF-1R and regulate NF-κB and Akt signaling, thereby influencing inflammatory gene expression, including IL-8, as well as epithelial-cell survival (47). These findings indicate that local IGF signaling can simultaneously support epithelial regeneration and modulate inflammatory responses, providing a mechanistic explanation for why intestinal IGF activity may differ from the systemic suppression observed in patients with active disease.

The effects of the IGF axis also extend beyond epithelial cells to the immune and stromal compartments. IGF-1 can promote an anti-inflammatory monocyte phenotype associated with increased IL-10 production, thereby suppressing intestinal inflammation (48). Similarly, IGF-2 has been implicated in macrophage metabolic reprogramming in experimental colitis, promoting oxidative phosphorylation and an anti-inflammatory phenotype (49). These observations suggest that IGF signaling may influence immune function not only through direct regulation of inflammatory pathways but also through changes in cellular metabolism. Stromal cells provide an additional layer of regulation. Mesenchymal stromal cells can ameliorate experimental colitis through an IGFBP-7-dependent immunosuppressive mechanism that limits T-cell proliferation and inflammatory cytokine production (50). Together, these findings support a model in which IGF-related signals mediate communication among epithelial, immune, and stromal cells and thereby coordinate tissue repair with resolution of inflammation.

IGFBPs further contribute to this local regulatory network and should therefore be considered active modulators rather than merely carriers of IGFs. IGFBP-3 levels are reduced during active IBD, whereas experimental supplementation or overexpression of IGFBP-3 suppresses aberrant crypt formation and colitis-associated tumor development without substantially changing the underlying severity of intestinal inflammation (51). This finding suggests that IGFBP-3 may influence inflammation-associated carcinogenesis through mechanisms that are at least partly distinct from direct control of intestinal inflammation. However, reduced circulating IGFBP-3 should not be taken to indicate that IGFBP-3 deficiency impairs mucosal repair. Circulating levels may not reflect IGFBP-3 activity within the mucosa, and IGFBP-3 can be detrimental in some settings: IGFBP-3 knockout mice were protected from DSS-induced colitis, with increased epithelial proliferation (52).

While more than 90% of IGFBP-3 is bound in a complex with IGF-1 in blood circulation (53), the other IGFBP-3 can also act independently of IGFs by binding to transmembrane protein 219 (TMEM219) (54) or low-density lipoprotein receptor-related protein 1 (LRP-1) (55, 56) at the tissue site. TMEM219 binds IGFBP-3 to actively mediate apoptosis, autophagy, and caspase activation (57–59). In inflammatory contexts, this pathway can regulate the lifespan of immune cells or damaged tissue cells (58).

In CD, expression levels of intestinal TMEM219 and IGFBP-3 are increased in active disease, and TMEM219 signaling is linked to caspase-8 activation and intestinal stem cell death. Blockade of IGFBP-3/TMEM219 binding with the recombinant TMEM219 ectodomain restored self-renewal of organoids from patients with CD *in vitro* and ameliorated DSS-induced and T cell-mediated colitis in mice, whereas IGF-1 did not rescue the IGFBP-3-induced loss of organoid growth (59). LRP-1 functions as an IGFBP-3 receptor, mediating IGF-independent growth inhibition through IRS-1/2 signaling (55, 56). IGFBP-3-LRP-1 signaling can activate IRS-2-PP2A-p130/p107, promoting epithelial growth arrest/tumor-suppressive signaling (60). LRP-1 also mediates IGFBP-3 endocytosis and clearance; loss of LRP-1 core fucosylation reduces IGFBP-3 uptake and increases circulating IGFBP-3 (61). Internalized IGFBP-3 can translocate to the nucleus where it has been reported to interact with transcription factors like RXR-α to modulate gene expression related to cell survival and inflammation (62, 63). These LRP-1 findings derive mainly from *in vitro* cell systems and non-intestinal models, and their relevance to IBD remains to be established.

Other IGFBPs exhibit additional context-dependent activities. IGFBP-5 expression is reduced in UC through epigenetic repression involving CTCF/EZH2-dependent H3K27me3 modification, while IGFBP-5 supplementation inhibits NF-κB activation and epithelial injury (32). IGFBP-7 produced by mesenchymal stromal cells can exert immunosuppressive effects by limiting T-cell proliferation and inflammatory cytokine production (50). Thus, the biological consequences of IGF signaling cannot be predicted solely from the circulating concentration of IGF-1 or IGFBPs; they are also shaped by the local expression and activity of individual IGFBPs including their IGF-independent receptor signaling and their interactions with epithelial, immune, and stromal cells.

### 3.3 Context-dependent IGF signaling in fibrosis, carcinogenesis, and therapeutic intervention

The reparative effects of IGF signaling may become maladaptive when signaling is sustained during chronic inflammation. In experimental colitis, IGF-1 stimulates collagen synthesis in colonic smooth muscle cells through an IGFBP-dependent mechanism, increasing extracellular-matrix deposition (64). Because IGF-1 can simultaneously enhance collagen production and reduce collagenase activity, persistent activation of this pathway may contribute to extracellular-matrix accumulation and potentially to the development of intestinal fibrosis and strictures. These findings illustrate an important aspect of IGF biology in IBD: a pathway that promotes tissue repair in the acute phase may contribute to pathological remodeling when activated chronically.

A similar context dependence may apply to carcinogenesis. In UC, IGF-1R expression in epithelial cells increases during mild inflammation and may promote the survival and proliferation of epithelial cells carrying genetic damage, potentially contributing to carcinogenic progression during chronic disease (65, 66). Consistent with this possibility, IGF-1R haploinsufficiency in mice improves mitochondrial function, reduces oxidative stress, and suppresses the development of colitis-associated colorectal cancer (67). Although these experimental findings do not establish that excessive IGF signaling directly drives colorectal cancer in patients with IBD, they suggest that prolonged IGF-1R activation under inflammatory conditions may have consequences that differ substantially from its beneficial effects on epithelial repair.

The context-dependent nature of IGF signaling also has implications for therapeutic development. Experimental approaches designed to deliver IGF-1 locally have shown potential benefits while limiting the need for broad systemic activation. IGF-1-coated sutures improve anastomotic healing and collagen deposition in inflamed intestine (68), whereas recombinant *Lactococcus lactis* engineered to express IGF-1 reduces the severity of DSS-induced colitis and improves epithelial integrity, providing proof of concept for microbiota-based local delivery (69). Conversely, clinical observations indicate that restoration of the endogenous IGF axis may occur as inflammation is effectively controlled. In patients with CD, infliximab treatment normalizes serum IGF-1 and IGFBP-3 levels, linking successful anti-inflammatory therapy with recovery of systemic IGF signaling (44). Corticosteroid treatment similarly improves, although does not completely normalize, abnormalities in the IGF system (41).

Taken together, these findings support a model in which the IGF axis functions as a dynamic interface between inflammation, metabolism, epithelial repair, immune regulation, and tissue remodeling in IBD. Systemic IGF suppression during active disease is associated with catabolism and impaired growth, whereas local IGF signaling can promote epithelial regeneration and anti-inflammatory immune responses. At the same time, persistent activation of specific IGF pathways may contribute to fibrosis or carcinogenesis. Therefore, future therapeutic strategies should consider not simply whether IGF signaling should be increased or inhibited, but rather how its activity can be modulated according to the relevant tissue compartment, cellular target, disease stage, and specific IGF/IGFBP pathway involved, including IGF-independentIGFBP-3 signaling through TMEM219.

## 4 GLPs in IBD: emerging roles in immunometabolism, intestinal repair, and therapeutic potential

From a mechanistic perspective, GLP signaling may influence IBD through multiple pathways, including modulation of inflammatory signaling, repair of the epithelial barrier, immunomodulation, and regulation of the microbiome. GLP-1 has been shown in experimental studies to suppress inflammatory signaling pathways and reduce the production of cytokines involved in intestinal inflammation, including TNFα and various interleukins regulated by NF-κB. Meanwhile, GLP-2 contributes to intestinal growth and repair primarily by promoting epithelial cell proliferation, enhancing nutrient absorption, and maintaining mucosal integrity (70). A review of the biological roles of GLP in IBD suggests that GLP-based therapies and DPP-4 inhibitors may have potential as immunometabolic approaches to intestinal inflammation; however, the clinical evidence remains preliminary (71).

Accumulating evidence linking metabolic dysfunction, obesity, and IBD provides an additional rationale for investigating GLP-1 receptor agonists (GLP-1 RAs) in patients with IBD. Obesity is associated with adipose tissue dysfunction, chronic low-grade inflammation, gut microbiota imbalance (dysbiosis), and impaired epithelial barrier function. In CD, expansion of mesenteric adipose tissue is associated with transmural inflammation and fibrosis (72). Because GLP-1 RAs have established effects on body weight and metabolic control, they may provide indirect benefits for IBD by improving obesity-associated metabolic and inflammatory disturbances. In addition, experimental studies suggest that GLP-1 signaling may have direct effects on intestinal inflammatory responses, epithelial integrity, and the gut microbiota (19). However, these mechanistic findings are derived predominantly from preclinical studies and should be distinguished from evidence of clinical efficacy in human IBD.

Preclinical studies provide substantial mechanistic support for GLP-based pathways in intestinal inflammation. Experimental colitis models have demonstrated that GLP-1 RAs can reduce intestinal inflammation, preserve epithelial integrity, improve barrier function, and regulate inflammatory immune responses. GLP-2 analogs also promote epithelial proliferation and intestinal regeneration, supporting their potential role in intestinal repair. DPP-4 inhibition and GLP-2 analog treatment have similarly shown protective effects in experimental models (24, 71). These findings provide biological plausibility for GLP-based interventions but do not establish that these therapies directly modify intestinal inflammation or induce mucosal healing in patients with IBD. Limited human evidence for GLP-2 analogs, including teduglutide, remains insufficient to establish clinical efficacy (24, 71).

Human evidence regarding GLP-1 RAs in IBD is currently derived primarily from observational studies. A nationwide Danish cohort study involving patients with both IBD and type 2 diabetes found that treatment with GLP-1 receptor agonists or DPP-4 inhibitors was associated with a lower risk of adverse IBD outcomes, including corticosteroid use, TNFα inhibitor treatment, hospitalization, and surgery, compared with other antidiabetic medications (adjusted IRR: 0.52, 95% CI: 0.42–0.65) (73). Because GLP-1 receptor agonists and DPP-4 inhibitors were evaluated together, these observational findings are consistent with a potential beneficial association between incretin-based therapy and IBD outcomes, but they do not establish a direct anti-inflammatory effect of GLP-1 receptor activation. Improved metabolic status, weight loss, altered microbiota, medication-related effects, or residual confounding may also contribute. Similarly, a nationwide Israeli cohort study reported that GLP-1 analog use was associated with a lower risk of composite adverse outcomes in patients with both UC and CD, with a stronger association observed among patients with obesity (74). Although these findings are encouraging, they remain observational and cannot determine whether the reported benefits result from direct intestinal effects, improvements in obesity and metabolic health, or other patient-related factors.

Recent systematic reviews further support the metabolic benefits and apparent tolerability of GLP-1 RAs in patients with IBD. A systematic review and meta-analysis of eight retrospective cohort studies involving 1,236 patients found that GLP-1 RA treatment was associated with significant reductions in body weight and BMI, although the certainty of evidence was low and heterogeneity among studies was substantial (75). Another systematic review of 14 studies reported reductions in body weight and improvements in metabolic markers, while available observational data did not indicate an increased risk of IBD exacerbation. Several studies also reported associations with reduced corticosteroid use, hospitalization, and surgery (76). However, because most of the available studies were retrospective, these findings should be interpreted as associations rather than evidence that GLP-1 RAs directly suppress intestinal inflammation or promote mucosal healing.

Overall, current evidence suggests that GLP-1 and GLP-2 pathways are biologically plausible targets for further investigation in IBD, but their therapeutic roles remain incompletely established. The evidence is strongest for the metabolic effects of GLP-1 RAs and for the intestinal reparative effects of GLP-2 in preclinical models. Although observational studies have reported favorable IBD-related outcomes among GLP-1 RA users, prospective clinical evidence demonstrating direct effects on intestinal inflammation, mucosal healing, or steroid-free remission remains lacking (23, 24, 77). Prospective randomized clinical trials in patients with active IBD are therefore needed to determine whether GLP-based therapies exert disease-modifying effects beyond their metabolic benefits and to clarify which patients may derive the greatest clinical benefit.

## 5 Effects of IBD on insulin signaling

The relationship between IBD and insulin signaling is bidirectional. Although insulin resistance is well established in DM, whether IBD itself causes persistent impairment of insulin sensitivity remains unclear. Early studies reported increased fasting insulin levels and insulin resistance in patients with IBD, whereas subsequent larger studies found that insulin resistance was more strongly associated with obesity, abdominal adiposity, hypertriglyceridemia, and nonalcoholic fatty liver disease than with IBD activity or phenotype (78). Importantly, a hyperinsulinemic-euglycemic clamp study in patients with active ulcerative colitis demonstrated reduced insulin sensitivity that improved following resolution of intestinal inflammation, suggesting that inflammatory activity can induce a transient and reversible impairment of insulin signaling (25). Thus, metabolic abnormalities in IBD likely reflect the combined effects of systemic inflammation and conventional metabolic risk factors rather than a uniform, intrinsic defect in insulin signaling.

Several mechanisms may connect intestinal inflammation with systemic insulin signaling. Proinflammatory cytokines and adipose-derived mediators can interfere with insulin-responsive metabolic pathways, while alterations in lipid metabolism and the gut microbiota may further influence insulin sensitivity. Consistent with this concept, studies of anti-TNF therapy have reported variable improvements in insulin sensitivity, with greater effects observed in some patients without major metabolic comorbidities (79, 80). In addition, reduced levels of the anti-inflammatory adipokine CTRP3 have been associated with greater insulin resistance and inflammatory activity in IBD (81). Dietary and metabolic interventions that improve insulin sensitivity have likewise been associated with reduced inflammatory responses in experimental and clinical settings. Together, these findings suggest that intestinal inflammation, adipose-derived signals, diet, and systemic metabolism form an interconnected network linking IBD to insulin signaling.

This bidirectional relationship may be particularly relevant in patients with IBD and DM, in whom pre-existing metabolic dysfunction may interact with inflammation to amplify systemic insulin resistance. Conversely, effective control of intestinal inflammation may partially restore insulin sensitivity in patients whose metabolic abnormalities are inflammation driven. Therefore, rather than viewing DM as a separate comorbidity, the IBD-DM relationship provides a clinically relevant context for understanding how inflammatory and metabolic signals converge on insulin pathways. Further studies are needed to determine whether improving insulin sensitivity can independently modify intestinal inflammation and whether metabolic phenotyping can identify IBD patients most likely to benefit from insulin-targeted interventions.

## 6 Personal medicine between IBD and diabetes

A potentially complex and bidirectional association exists between IBD, including CD and UC, and diabetes. While growing evidence indicates higher prevalence and incidence rates of diabetes among patients with IBD, diabetes, particularly type 2 diabetes (T2DM), may also increase the subsequent risk of developing IBD. This association is thought to reflect the interplay of chronic systemic inflammation, insulin resistance, intestinal barrier dysfunction, alterations in gut microbiota, immune dysregulation, and metabolic abnormalities. Importantly, although observational studies consistently demonstrate a link between IBD and diabetes, recent genetic studies have yielded conflicting results regarding causality, suggesting that this relationship is multifactorial rather than stemming from a single pathogenic pathway.

A meta-analysis by Li et al. demonstrated that IBD is associated with a significant increase in the risk of diabetes, with a pooled odds ratio (OR)/relative risk (RR) of 1.26 (95% confidence interval: 1.03–1.53). This increased risk was observed for both UC (OR/RR 1.33) and CD (OR/RR 1.39). The analysis also revealed an increased risk of ischemic heart disease among IBD patients, suggesting that the link between IBD and diabetes may not be merely a localized gastrointestinal-metabolic interaction but rather part of a broader cardiometabolic phenotype (82). Consistent with these findings, a large-scale study using UK Biobank data, involving over 400,000 participants, reported an increased risk of T2DM in patients with CD (hazard ratio [HR] 2.11) and UC (HR 1.76), as well as associations between IBD and several other cardiometabolic diseases (83). Interestingly, this epidemiological association may be bidirectional. A prospective cohort study of 313,008 UK Biobank participants, followed for a median of 13.72 years, showed that individuals with pre-existing T2DM had a higher incidence of IBD compared to those without diabetes. After multivariate adjustment, T2DM was associated with an increased risk of developing IBD (HR 1.61), CD (HR 2.10), and UC (HR 1.58), and these associations remained significant even after propensity score matching (84).

On the other hand, genetic evidence suggests a need for a more cautious interpretation regarding the association between IBD and T2DM. Two-sample Mendelian randomization (MR) studies found no significant causal effect of IBD on T2DM or vice versa, thereby ruling out a simple, genetically mediated causal relationship (85). Similarly, while the aforementioned UK Biobank study observed an association between IBD and cardiometabolic diseases in observational analyses, genetic and two-sample MR analyses failed to demonstrate a consistent link (83). In contrast, another MR study involving European populations reported a statistically significant, albeit extremely small, effect (odds ratio: approximately 1.02-1.03) of IBD (UC and CD) on T2DM, whereas no significant association was observed in East Asian populations (86). These discrepancies across studies may reflect differences in population-specific genetic backgrounds, environmental exposures, the genetic instruments used for MR, or residual horizontal pleiotropy. Thus, while epidemiological evidence strongly supports an association between IBD and T2DM, current genetic evidence has not established a consistent, direct causal pathway. This relationship likely reflects complex interactions among genetic susceptibility, chronic inflammation, metabolic factors, environmental exposures, and the gut microbiota. The comorbidity of IBD and T2DM is clinically significant, as it may increase the systemic and healthcare-related burdens associated with these diseases. A systematic review and meta-analysis involving 71,216 patients revealed that IBD patients with comorbid diabetes face a significantly higher risk of IBD-related hospitalization (OR: 2.52) and sepsis (OR: 1.56), as well as an increased risk of pneumonia and urinary tract infections. Furthermore, patients with both conditions exhibited lower QOL scores and substantially higher annual medical costs compared to those with IBD alone (87). However, the analysis found no significant association between diabetes and IBD-related complications, surgeries, or mortality rates. Similarly, a recent case-control study reported comparable rates of severe IBD (59.5% vs. 59.7%) between IBD patients with and without T2DM, suggesting that T2DM does not necessarily exacerbate the severity of intestinal inflammation itself (88). Conversely, the coexistence of these two diseases is associated with a markedly higher frequency of hepatic steatosis and liver injury, highlighting the importance of metabolic and extra-intestinal complications in patients with both conditions (88).

The association between IBD and diabetes extends to type 1 diabetes (T1DM), although the underlying biological mechanisms may differ significantly from those involved in T2DM. Both T1DM and IBD are immune-mediated diseases, and their co-occurrence may reflect shared abnormalities in immune regulation and genetic susceptibility. A large pediatric cohort study involving 65,147 patients with T1DM found that approximately 0.1% also had IBD. Children with both T1DM and IBD tended to have a lower BMI and a higher frequency of severe hypoglycemia compared to those with T1DM alone; this may reflect intestinal inflammation, malabsorption, nutritional impairment, and alterations in glycemic control (89). A subsequent nationwide study in Sweden demonstrated a bidirectional epidemiological association between IBD and T1DM. Patients with IBD were at an increased risk of subsequently developing T1DM (adjusted hazard ratio [aHR] 1.58), with a particularly strong association observed in patients with UC (aHR 2.02). Conversely, a higher prevalence of a history of T1DM was found among patients with IBD (adjusted odds ratio [aOR] 1.36) (90). These findings support the existence of a common immune-mediated pathway linking intestinal inflammation to autoimmune destruction of pancreatic β-cells.

Collectively, current evidence suggests that the relationship between IBD and diabetes is complex and varies by disease type. While an association between IBD and T2DM is consistently observed in epidemiological studies and appears to be bidirectional, Mendelian randomization analyses have not yielded consistent evidence of a direct causal link. This suggests that the observed association may arise from a combination of factors, including chronic inflammation, insulin resistance, alterations in the gut microbiota, intestinal barrier dysfunction, environmental factors, and shared metabolic susceptibility. In contrast, the association between IBD and T1DM may rest on a stronger immunological foundation, given that both conditions are characterized by dysregulation of adaptive and innate immune responses. Importantly, the coexistence of IBD and diabetes appears to increase the risk of systemic complications, infections, healthcare utilization, and metabolic comorbidities, even in the absence of significantly increased intestinal disease severity. Future research integrating genetic, immunological, metabolic, and microbiome data will be crucial to determine whether specific inflammatory or metabolic pathways linking IBD and diabetes could serve as therapeutic targets.

## 7 Conclusion

Current evidence supports a close association and biologically plausible bidirectional relationship between metabolic signaling and intestinal inflammation in IBD. Insulin, IGF, and GLP pathways participate in the regulation of epithelial integrity, immune responses, tissue repair, and systemic metabolism, but their effects are highly dependent on cellular compartment, disease state, and metabolic context. Thus, these pathways should not be viewed as uniformly protective or pathogenic. Rather, they represent interconnected components of an immunometabolic network in which intestinal inflammation can alter systemic metabolic signaling, while metabolic signals can in turn influence intestinal homeostasis and disease-associated remodeling.

Several important questions remain unresolved. It is unclear which effects of insulin, IGF, and GLP signaling are directly mediated within the intestinal mucosa and which reflect systemic metabolic changes. The relative contributions of epithelial, immune, stromal, and metabolic tissues also remain incompletely defined. In addition, although observational and experimental studies suggest potential benefits of manipulating insulin- and GLP-related pathways, it remains uncertain whether these effects translate into direct disease modification, sustained mucosal healing, or improved long-term outcomes in IBD.

Future research should therefore integrate metabolic, immunologic, microbiome, and tissue-specific approaches to define the mechanisms and cellular contexts that determine the effects of these pathways. Prospective clinical trials, particularly those incorporating disease phenotype, inflammatory activity, and metabolic status, will be essential to determine whether targeting insulin, IGF, or GLP signaling can provide therapeutic benefit beyond conventional metabolic effects. A better understanding of the potential bidirectional interactions within this immunometabolic network may ultimately support more individualized strategies for managing IBD in patients with coexisting metabolic dysfunction.

## Glossary

AKT: protein kinase B (PKB)
ALS: acid-labile subunit
CD: Crohn’s disease
COX-2: cyclooxygenase-2
CRP: C-reactive protein
CTCF: CCCTC-binding factor
CTRP3: C1q/TNF-related protein 3
DM: diabetes mellitus
DNA: deoxyribonucleic acid
DPP-4: dipeptidyl peptidase-4
DSS: dextran sodium sulfate
ECM: extracellular matrix
ERK: extracellular signal-regulated kinase
EZH2: enhancer of zeste 2 polycomb repressive complex 2 subunit
GLP-1: glucagon-like peptide-1
GLP-1 RA: GLP-1 receptor agonist
GLP-2: glucagon-like peptide-2
H3K27me3: trimethylation of lysine 27 on histone H3 (tri-methyl-histone H3 at lysine 27)
HOMA-IR: homeostatic model assessment for insulin resistance
HR: hazard ratio
aHR: adjusted hazard ratio
IBD: inflammatory bowel disease
IGF-1R: IGF-1 receptor
IGF-2R: IGF-2 receptor
IGFBP-1: insulin-like growth factor binding protein-1
IGFBP-2: insulin-like growth factor binding protein-2
IGFBP-3: insulin-like growth factor binding protein-3
IGFBP-5: insulin-like growth factor binding protein-5
IGFBP-7: insulin-like growth factor binding protein-7
IGF-1: insulin-like growth factor-1
IGF-2: insulin-like growth factor-2
IL-6: interleukin-6
IL-8: interleukin-8
IL-10: interleukin-10
IL-17: interleukin-17
IR: insulin receptor
IRR: incidence rate ratio
IRS: insulin receptor substrate
LRP1: low-density lipoprotein receptor-related protein 1
MAPK: mitogen-activated protein kinase
MR: mendelian randomization
NAFLD: non-alcoholic fatty liver disease
NF-κB: nuclear factor kappa-light-chain-enhancer of activated B cells
OR: odds ratio
aOR: adjusted odds ratio
PI3K: phosphatidylinositol 3-kinase
PPARγ: peroxisome proliferator-activated receptor gamma
PWV: pulse wave velocity
QOL: quality of life
ROS: reactive oxygen species
RR: relative risk
RXR-α: Retinoid X Receptor-alpha
SULT1A1: sulfotransferase family 1A member 1
SULT1A3: sulfotransferase family 1A member 3
T1DM: type 1 diabetes mellitus
T2DM: type 2 diabetes mellitus
TGR5: Takeda G protein-coupled receptor 5
TMEM219: transmembrane protein 219
TNFα: tumor necrosis factor alpha
TRM: tissue-resident memory T cells
UC: ulcerative colitis
UK: United Kingdom
VPS24: vacuolar protein-sorting-associated protein 24
